# Draft genome sequences of *Staphylococcus hominis* strains O226 and O218 and *Micrococcus luteus* O220 isolated from the buccal swabs of healthy individuals

**DOI:** 10.1128/mra.00810-25

**Published:** 2025-09-26

**Authors:** Sandra Jablonska, Lila Nelson, Grace Finger, Alex Kula, Catherine Putonti

**Affiliations:** 1 Bioinformatics Program, Loyola University Chicago 2456, Chicago, Illinois, USA; 2 Biology Department, Loyola University Chicago 2456, Chicago, Illinois, USA; University of Wisconsin-Madison, Madison, Wisconsin, USA

**Keywords:** *Staphylococcus hominis*, *Micrococcus luteus*, buccal, oral microbiome

## Abstract

To investigate the genomic features of rare oral commensals in healthy individuals, we present the draft genome sequences of *Staphylococcus hominis* strains O226 and O218 and *Micrococcus luteus* strain O220. All three of these isolates were purified from buccal swab samples collected from healthy female participants.

## ANNOUNCEMENT

*Staphylococcus* and *Micrococcus* are both common genera of the healthy human oral microbiome (1). Although neither *Staphylococcus hominis* nor *Micrococcus luteus* is a predominate member of this community, both have been shown to produce antimicrobial compounds and thus may play a role in maintaining a “healthy” community (2, 3). Recently, we conducted a study to isolate members of the buccal mucosa of healthy individuals, and here we present the draft genome sequences of two *S*. *hominis* isolates and one *M. luteus* isolate.

Participants in our institutional review board (IRB)-approved study (Loyola University Chicago, IRB no. 3603) self-identified as female, “healthy,” and had not taken antibiotics within the last 6 months. The three isolates described here are from different individuals. Each participant was instructed to rub the cotton swab (BD BBL CultureSwab) on their inner cheek for 30 seconds, after which the swab was inserted into the provided storage solution (Amies media). Within 1 hour of sampling, swabs were swirled and squeezed in the storage media, diluted 100× in 0.5% saline, spread on mannitol salt (MS) agar plates, and incubated for 24 hours at 35°C with 5% CO_2_. Morphologically distinct colonies were selected and grown in MS broth under the same conditions. This process was repeated to purify the isolates. DNA was extracted using the DNeasy Blood and Tissue kit (Qiagen), following the manufacturer’s protocol for Gram-positive bacteria, and quantified via the Qubit fluorometer. SeqCoast Genomics (Portsmouth, NH, USA) performed library construction using the Illumina DNA Prep tagmentation kit and unique dual indexes and sequencing via the Illumina NextSeq2000 platform using a 300-cycle flow cell kit (2 × 150 bp reads). Read demultiplexing, read trimming, and run analytics were performed using DRAGEN v.3.10.12 prior to delivery.

The BV-BRC v.3.51.7 web tool was used to generate the draft genome assemblies (4). Using the “auto” assembly strategy parameter, raw reads were first trimmed using trim_galore v.0.6.5dev (https://github.com/FelixKrueger/TrimGalore) and then normalized via bbnorm v.37.60 (https://sourceforge.net/projects/bbmap/). Next, reads were assembled using Unicycler v.0.4.8 (5), followed by two rounds of polishing by Pilon v.1.24 (6). Genomic coverage was calculated by BV-BRC. Taxonomic identification was first predicted via BV-BRC’s taxonomic binning tool (based on Kraken 2 (7)) and then confirmed by JSpeciesWS (8); average nucleotide identity (ANI) was calculated for each draft genome and genomes for the predicted species in the JSpeciesWS database. Genome annotations were generated using the NCBI Prokaryotic Genome Annotation Pipeline (PGAP) v.6.10 (9). Completeness and contamination metrics were computed using CheckM v.1.2.3 (10) upon submission to NCBI. Secondary metabolites were predicted using antiSMASH v.8 (11). Default parameters were used for all software tools unless otherwise noted.

Table 1 lists the assembly statistics for the three genomes. *M. luteus* O220 shared 97.80% ANI with *M. luteus* ATCC 4698 (GCF_006094415.1). *S. hominis* strains O218 and O226 shared 98.76% and 99.86% ANI, respectively, with *S. hominis subsp. hominis* C80 (GCF_000183685.1). Multiple secondary metabolites with potential antimicrobial properties were identified for all three genome assemblies.

**TABLE 1:** Genome assembly statistics for *M. luteus* O220 and *S. hominis* strains O218 and O226

Strain	O220	O218	O226
Species designation	*M. luteus*	*S. hominis*	*S. hominis*
Assembly length (bp)	2,531,396	2,280,898	2,153,502
No. of contigs	106	33	38
Contigs N50 (bp)	47,294	189,100	219,789
Coverage (×)	289.03	217.15	426.91
# genes	2,383	2,302	2,205
# tRNAs	48	50	48
G + C (%)	71.7	32.4	32.3
Completeness (%)	98.7	92.69	90.2
Contamination (%)	2.76	2.93	2.41
No. of raw reads	8,453,942	3,356,184	5,804,424
No. of reads after trimming	8,450,892	3,355,380	5,803,106
No. of reads after normalization (used for assembly)	3,844,060	2,242,528	2,551,620
SRA accession no.	SRR34298191	SRR34298192	SRR34298190
Assembly accession no.	JBPFAN00000000	JBPFAM000000000	JBPFAO000000000
Predicted secondary metabolite regions (contig no.)^*a*^	RRE-containing (8); NAPAA (10); Ectoine (11); NI-siderophore (21); Terpene-precursor (22); Terpene (30); Betalactone (45)	Terpene-precursor (2); T3PKS (4); NI-siderophore (7); Class II lanthipeptide (16); Cyclic lactone autoinducer (18)	Terpene-precursor (3); T3PKS (4); NI-siderophore (6); Cyclic lactone autoinducer (8)

^*a*^ AntiSMASH metabolite abbreviations: Betalactone: beta-lactone containing protease inhibitor; Cyclic lactone autoinducer: *agrD*-like cyclic lactone autoinducer peptides; NAPAA: non-alpha poly-amino acids like e-Polylysin; NI-siderophore: non-ribosomal peptide synthetase (NRPS)-independent, *lucA*/*lucC*-like siderophores; T3PKS: type III PKS (polyketide synthase).

## Data Availability

Accession numbers for the deposited raw reads and annotated assembly can be found in Table 1.
